# A causal association between lipid-lowering medications and rotator cuff syndrome: a drug-targeted mendelian randomization study

**DOI:** 10.3389/fgene.2024.1383646

**Published:** 2024-06-06

**Authors:** Meng-meng Liu, Xiang Chen, Chuan-wen Yu, Jin-wei Chen, Pu-xiang Zhen, Zhi-peng Liu

**Affiliations:** ^1^ School of Physical Education and Health, Guangxi Medical University, Nanning, China; ^2^ Department of Bone and Joint Surgery, The First Affiliated Hospital of Guangxi Medical University, Nanning, China; ^3^ School of Physical Education and Health, Heze University, Heze, China; ^4^ Department of Physical Education, Dongshin University, Naju, Republic of Korea; ^5^ National Demonstration Center for Experimental (General Practice) Education, Hubei University of Science and Technology, Xianning, China; ^6^ Division of Hepatobiliary Surgery, The First Affiliated Hospital of Guangxi Medical University, Nanning, China

**Keywords:** rotator cuff syndrome, stains, LDL-C, TG, TC, mendelian randomization, drug target

## Abstract

**Background:** Previous research has suggested that dyslipidemia may be a risk factor for rotator cuff syndrome (RCS), and lipid-lowering drugs may aid in its treatment, though conclusions have not been definitive. Mendelian randomization is a statistical method that explores the causal relationships between exposure factors and diseases. It overcomes the confounding issues inherent in traditional observational studies, thereby providing more reliable causal inferences. We employed this method to investigate whether hyperlipidemia is a risk factor for rotator cuff syndrome and whether lipid-lowering drugs can effectively treat this condition.

**Methods:** Genetic variations linked to lipid traits low-density lipoprotein cholesterol (LDL-C), triglyceride (TG), and total cholesterol (TC) were acquired from the UK Biobank and the Global Lipids Genetics Consortium (GLGC). Data on genetic variation in rotator cuff syndrome were obtained from FinnGen, including 24,061 patients and 275,212 controls. In the next step, we carried out two-sample Mendelian randomization analyses to determine whether lipid traits correlate with rotator cuff syndrome risk. Additionally, we performed drug-target Mendelian randomization (MR) analyses on 10 drug targets related to rotator cuff syndrome. For the drug targets that showed significant results, further analysis was done using Summary-data-based Mendelian Randomization (SMR) and colocalization techniques. We performed a mediation analysis to identify potential mediators between HMG-CoA reductase (HMGCR) and RCS.

**Results:** No causative link was established between these lipid traits and rotator cuff syndrome. However, a significant association has been identified where HMGCR inhibition corresponds to a reduced risk of rotator cuff disease (OR = 0.68, [95% CI, 0.56–0.83], *p* = 1.510 × 10^−4^). Additionally, enhanced expression of HMGCR in muscle tissues is also linked to a decreased risk of rotator cuff syndrome (OR = 0.88, [95% CI, 0.76–0.99], *p* = 0.03). Body mass index (BMI) mediated 22.97% of the total effect of HMGCR on RCS.

**Conclusion:** This study does not support low-density LDL-C, TG, and TC as risk factors for rotator cuff syndrome. HMGCR represents a potential pharmaceutical target for preventing and treating rotator cuff syndrome. The protective action of statins on the rotator cuff syndrome might not be associated with their lipid-lowering properties.

## 1 Introduction

Rotator cuff syndrome (RCS), also known as rotator cuff tendinitis, manifests with intense pain and functional impairment (Roy et al., 2015). It is notably prevalent among athletes engaging in overhead activities, with reported incidence rates of 23% for handball players, 34% for baseball pitchers, and 35% for swimmers (Andersson et al., 2017; Lin et al., 2018; Davis et al., 2024). Among the general population, the incidence rate is 5%–10%, and it even exceeds 20% in those aged over 60 (Qian et al., 2023). Primary conservative interventions encompass local corticosteroid injections and oral nonsteroidal anti-inflammatory drugs (NSAIDs) (Desjardins-Charbonneau et al., 2015). Despite the short-term alleviation of pain demonstrated by corticosteroids and NSAIDs, their long-term efficacy remains uncertain while posing significant side effects (Andres and Murrell, 2008; Coombes et al., 2010). Thus, the search for new treatment medications is urgently required.

Observational studies have indicated that hyperlipidemia is a noteworthy risk factor for rotator cuff syndrome (Lin et al., 2015; Giri et al., 2023). Evidence suggests that patients with hyperlipidemia are at increased risk of recurrent tears following arthroscopic repair of the rotator cuff (Garcia et al., 2017). However, it is important to acknowledge the inherent limitations and potential confounding factors in epidemiological research. Consequently, the definitive causal relationship between hyperlipidemia and RCS has not yet been conclusively established.

Statins are widely accepted lipid-lowering drugs routinely used clinically for primary and secondary prevention of coronary heart disease (Lee et al., 2022). In the field of orthopedics, statins are also used for the prevention of tendon diseases (Teichtahl et al., 2016). A cohort study that spanned 11 years showed that statins provide benefits in preventing RCS in patients diagnosed with hyperlipidemia (Lin et al., 2015). Randomized controlled trials (RCTs) are the gold standard for assessing drug efficacy but are costly and challenging to conduct. There is a lack of high-quality RCTs examining lipid-lowering drugs in RCS. Thus, the link between these drugs and RCS remains inconclusive, necessitating further exploration.

Mendelian randomization (MR) utilizes genetic variants as instrumental variables to assess causal links between exposures and diseases, based on Mendel’s laws that suggest genetic variants are randomly distributed among individuals, similar to randomized controlled trials (RCTs). This approach mitigates confounding issues inherent in traditional observational studies, offering more robust causal inferences. Compared to RCTs, Mendelian randomization is less costly, quicker to implement, and can statistically analyze exposure factors that exacerbate disease risk, which cannot be ethically tested in RCTs (Sekula et al., 2016; Larsson et al., 2023). Drug target Mendelian randomization studies are a straightforward application of this method, where genetic variants encoding protein targets can affect the expression of target genes, and certain drugs can act on these targets, thus influencing gene expression (Schmidt et al., 2020; Ference, 2022). By using these genetic variants as proxies for exposure, the potential impact of these drug targets on disease risk can be estimated. To this end, we applied Mendelian randomization analysis to evaluate the causal relationship between lipid levels and RCS, and also examined the influence of lipid-lowering drug targets on this condition, aiming to identify preventive and therapeutic options.

### 1.1 Methods and materials

**FIGURE 1:**
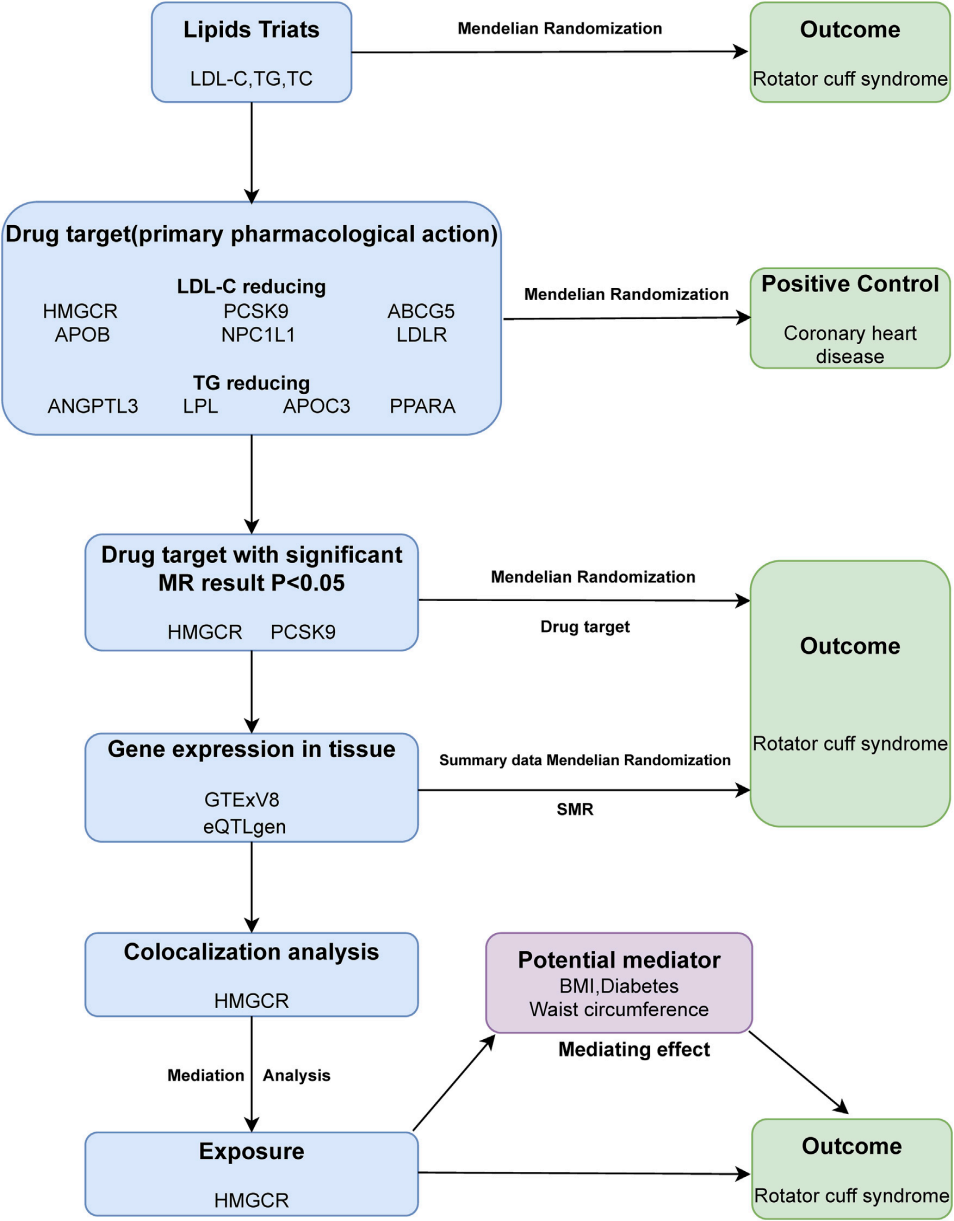
Outline of the study design.

**FIGURE 2:**
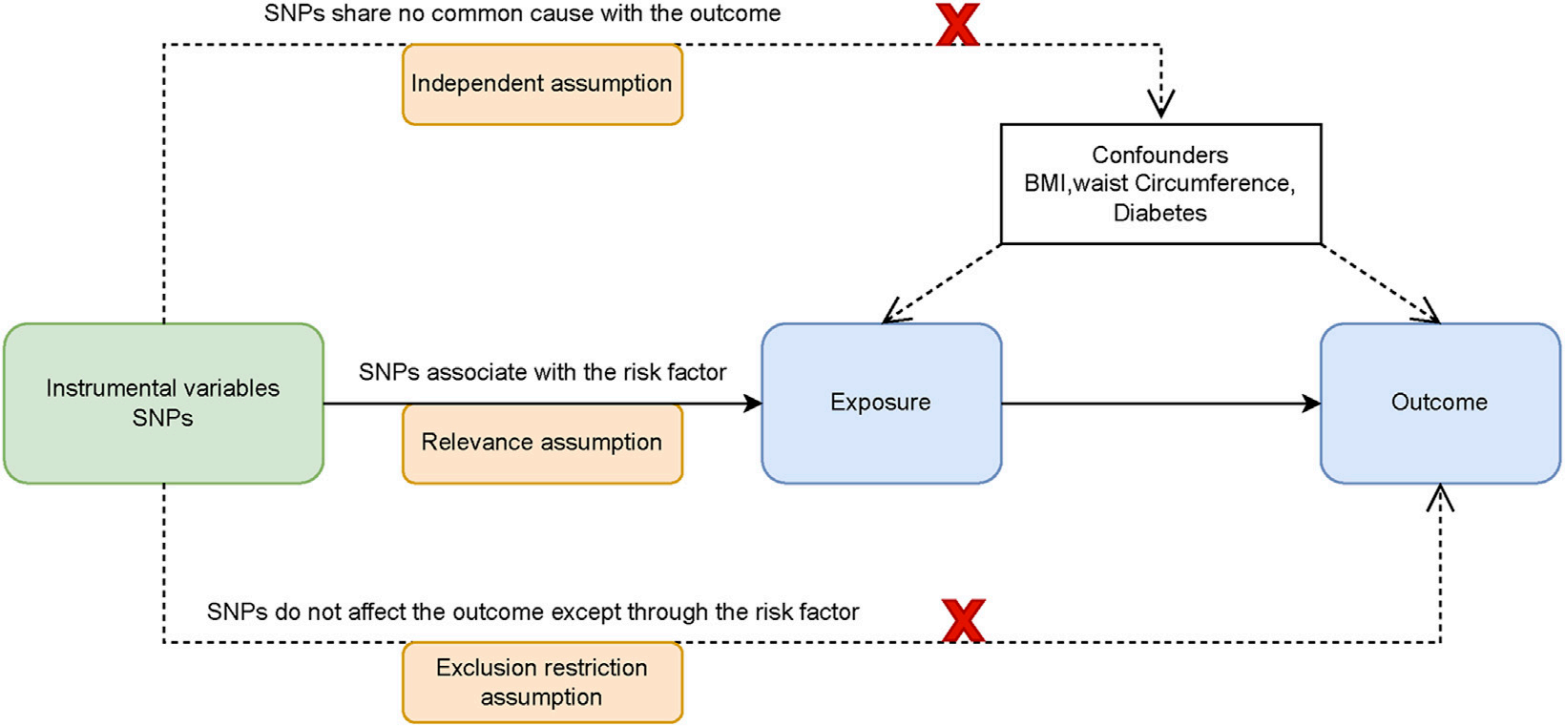
Three assumptions of Mendelian randomization.

Our study employs a three-step approach to explore the causal links between lipid traits and RCS. We begin with a two-sample Mendelian randomization analysis of three lipid traits to establish causality with the disease. This is followed by a drug-target analysis involving 10 genes associated with blood lipids to identify potential connections to the disease. SMR and colocalization analyses were conducted on the positive drug target genes, ultimately, a mediation Mendelian randomization analysis identified potential mediators of the relationship between HMGCR inhibition and RCS. The methodology is detailed in Figure 1. Mendelian randomization research must adhere to three fundamental presuppositions: (1) Relevance: Genetic variants are closely associated with exposure; (2) Independence: Genetic variants are not associated with confounders that influence the outcome; (3) Exclusivity: Genetic variants influence the outcome only through exposure (Figure 2).

In our study, we identified distinct instrumental variables related to lipid characteristics through a genome-wide association study (GWAS) conducted with data from the UK Biobank (Richardson et al., 2020) and the Global Lipids Genetics Consortium (GLGC) (Willer et al., 2013). These variables, specifically LDL-C (low-density lipoprotein cholesterol), TG (triglyceride), and TC (total cholesterol), were selected based on stringent criteria: a linkage disequilibrium (LD) clumping threshold of r^2^ < 0.001, *p* < 5 × 10^−8^, and a maximum physical distance of 10,000 kb between SNPs(single nucleotide polymorphisms). To refine our analysis and exclude SNPs potentially influenced by confounding factors such as BMI(body mass index), waist circumference, and diabetes (Cao et al., 2023; Giri et al., 2023), we utilized the PhenoScanner database (http://www.phenoscanner.medschl.cam.ac.uk/), resulting in the selection of 107, 175, and 71 SNPs as instrumental variables for LDL-C, TG, and TC, respectively. Additionally, we incorporated GWAS data on RCS sourced from the FinnGen(R9), applying the ICD-10 (International Classification of Diseases) standards for diagnosis. This dataset comprised 24,061 patients and 275,212 control subjects, all of European ancestry. Detailed information on all GWAS data can be found in Supplementary Table S1, while Supplementary Table S2 showcases the effective SNPs associated with the three lipid traits under investigation.

We leveraged the DrugBank database (https://go.drugbank.com/) to pinpoint genes encoding pharmacological targets of lipid-lowering drugs. These target genes were categorized based on their pharmacological impact into two groups: those that lower LDL-C (including PCSK9, ABCG5, APOB, HMGCR, NPC1L1, LDLR) and those that reduce TG levels (comprising ANGPTL3, LPL, APOC3, and PPARA). To approximate the effects of drug-target interactions, we employed a linkage disequilibrium (LD) clustering approach. Specifically, we identified variants within a ±100 kb radius of each target gene that were associated with LDL-C levels at a genome-wide significance threshold (*p* < 5 × 10^−8^). These SNPs were then clustered based on an LD r^2^ ≤ 0.2 and a maximum physical distance of 250 kb. Subsequently, to assess the efficacy of these genetic variants as drug targets, Mendelian randomization studies were carried out with coronary heart disease (CHD) as the anticipated outcome. This served as a positive control analysis to confirm the effectiveness of these genetic variations in drug targeting.

To delve deeper into the associations identified, Summary-data-based Mendelian Randomization (SMR) analysis (Zhu et al., 2016) was leveraged for drug targets that demonstrated noteworthy outcomes. We utilized expression quantitative trait loci (eQTL) corresponding to the implicated genes as surrogate markers for the effect of lipid-modifying medications. The acquisition of eQTL data was from the eQTLGen Consortium and the GTEx Consortium V8 databases. Our investigation strictly used cis-eQTLs as instrumental variables, establishing a significance criterion of *p* < 5 × 10^−8^ and a linkage disequilibrium (LD) cut-off of r^2^ < 0.1. A pivotal element of our methodology was applying colocalization analysis to validate the assumptions underpinning our instrumental variables, affirming that the observed associations between the exposure and the outcome were not obscured by different genetic variants in LD. Two of the five hypotheses tested through this approach warrant special attention: H3, positing that the correlated traits arise from distinct causal variants; and H4, indicating a shared causal variant underlies the correlation. The probability of colocalization was computed as the quotient of H4 over the aggregate of H3 and H4 (H4/(H3 + H4)), with a ratio exceeding 80% signaling a confirmatory finding.

### 1.2 Statistical analysis

In our Mendelian Randomization analysis, we apply five distinct methods to determine the causal relationships, focusing primarily on Inverse Variance Weighted (IVW), MR Egger, and Weighted Median as the core techniques. To evaluate heterogeneity across studies, we utilize the Cochran Q test and MR Egger heterogeneity. The MR-Egger intercept is specifically used to explore the presence of pleiotropy within our data. Additionally, the MR-PRESSO (Verbanck et al., 2018) strategy is adept at pinpointing outliers and adjusting causal estimates accordingly after their removal. We use the Bonferroni correction method to refine our significance thresholds: for analyses concerning three lipid phenotypes, a *p*-value below 0.016 (0.05/3) is deemed significant, whereas for investigations targeting ten lipid-related genes, a *p*-value below 0.005 (0.05/10) is required for statistical significance. “Two-Sample MR”, and “coloc” in R (version 4.2.2) were used for all statistical analyses.

## 2 Results

### 2.1 The association between lipid traits and rotator cuff syndrome

**FIGURE 3:**
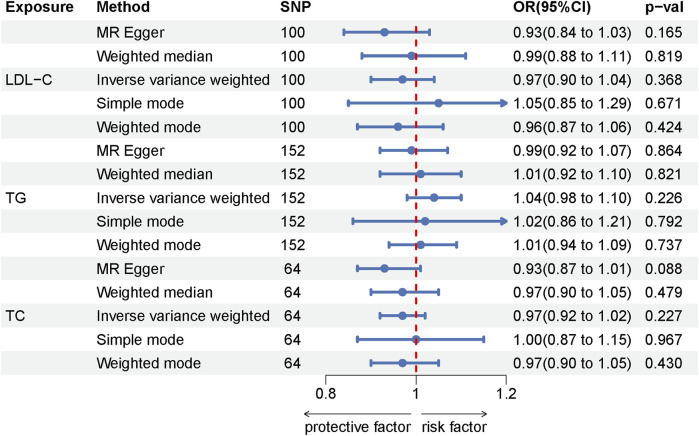
Forest plot of the MR results for the lipid-RCS.

Lipid characteristics, which include LDL-C, TG, and TC, are critical to the study of their association with RCS. Specifically, 107 independently related SNPs are linked to LDL-C, while TG and TC are associated with 175 and 71 independently related SNPs, respectively. All identified SNPs exhibit F-statistical analysis values above 10, ranging from 31 to 85,444, indicating strong instrument reliability. Instrumental variables reveal strong associations, supporting their use in Mendelian randomization analyses (Burgess et al., 2011). However, our investigation found no causal relationship between the three lipid traits (LDL-C, TG, TC) and RCS, as indicated in Supplementary Table S3. This conclusion is further supported by the absence of heterogeneity in the findings, as demonstrated by the Cochran Q test and MR-Egger heterogeneity analysis, and the lack of horizontal pleiotropy, confirmed by the MR-Egger intercept presented in Supplementary Table S4. These results reinforce the credibility of our research findings. For a visual representation of the Mendelian randomization outcomes, refer to the forest plot in Figure 3.

### 2.2 Association between lipid-lowering medication targets and rotator cuff syndrome

**FIGURE 4:**
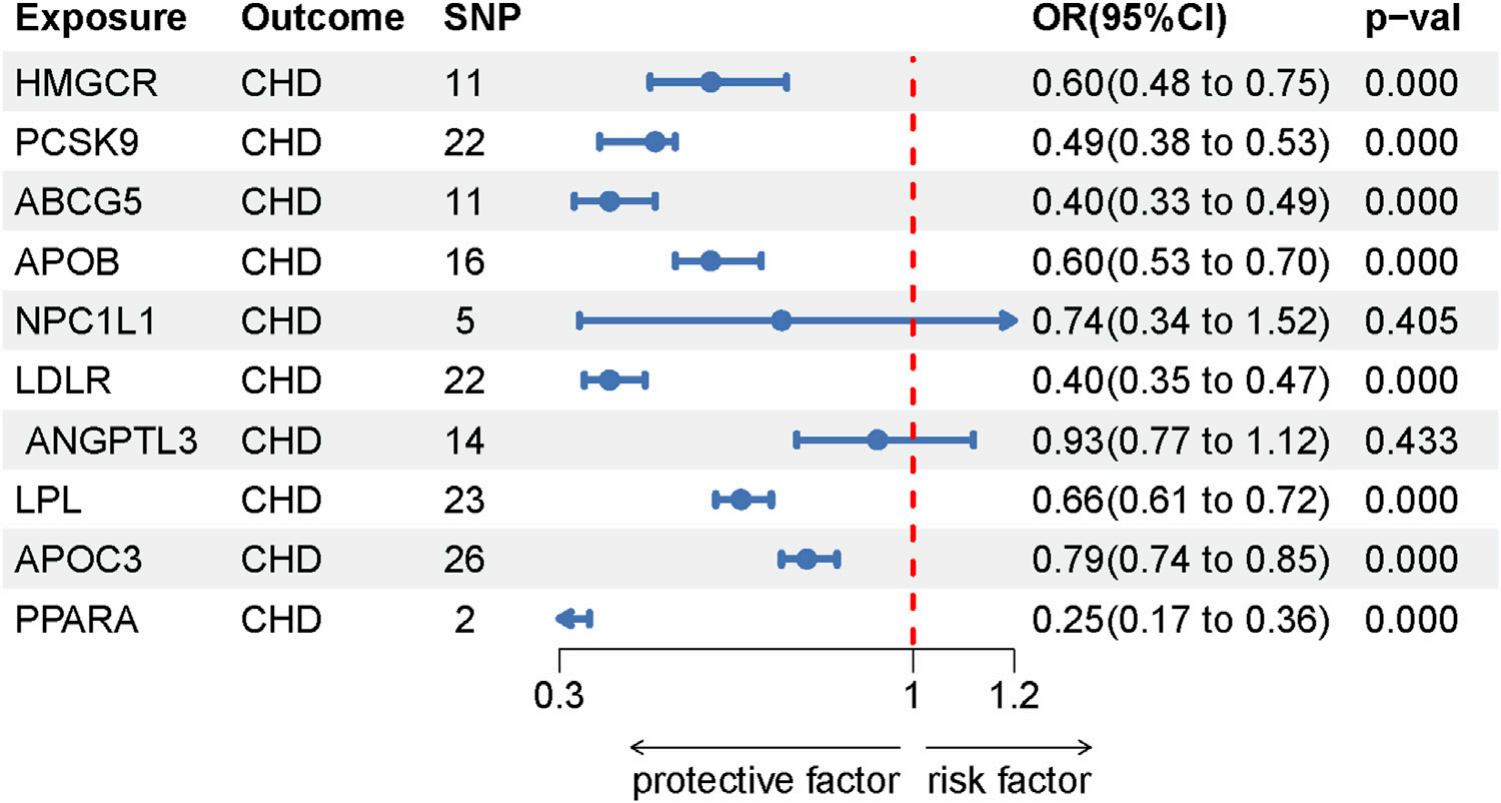
Forest plot of positive control IVW method.

This analysis integrates findings from two segments of our study, with the first focusing on identifying ten genes as potential targets for lipid-lowering medications: HMGCR, PCSK9, ABCG5, APOB, NPC1L1, LDLR, ANGPTL3, LPL, APOC3, and PPARA (Supplementary Table S5). The reliability of the selected SNPs for these genes is underscored by F-statistics exceeding the threshold of 10, indicating strong instrument validity (detailed data provided in Supplementary Table S6. In a positive control analysis, eight of these targets showed a significant link to a lower risk of coronary heart disease, underscoring the effectiveness of these genetic instruments. However, the association between NPC1L1, ANGPTL3, and coronary heart disease did not reach statistical significance, though a protective trend was observed. These positive control MR findings are documented in Supplementary Table S7, with heterogeneity and pleiotropy analyses detailed in Supplementary Table S8. The Mendelian randomization outcomes via the IVW method are visually represented in the forest plot of Figure 4.

**FIGURE 5:**
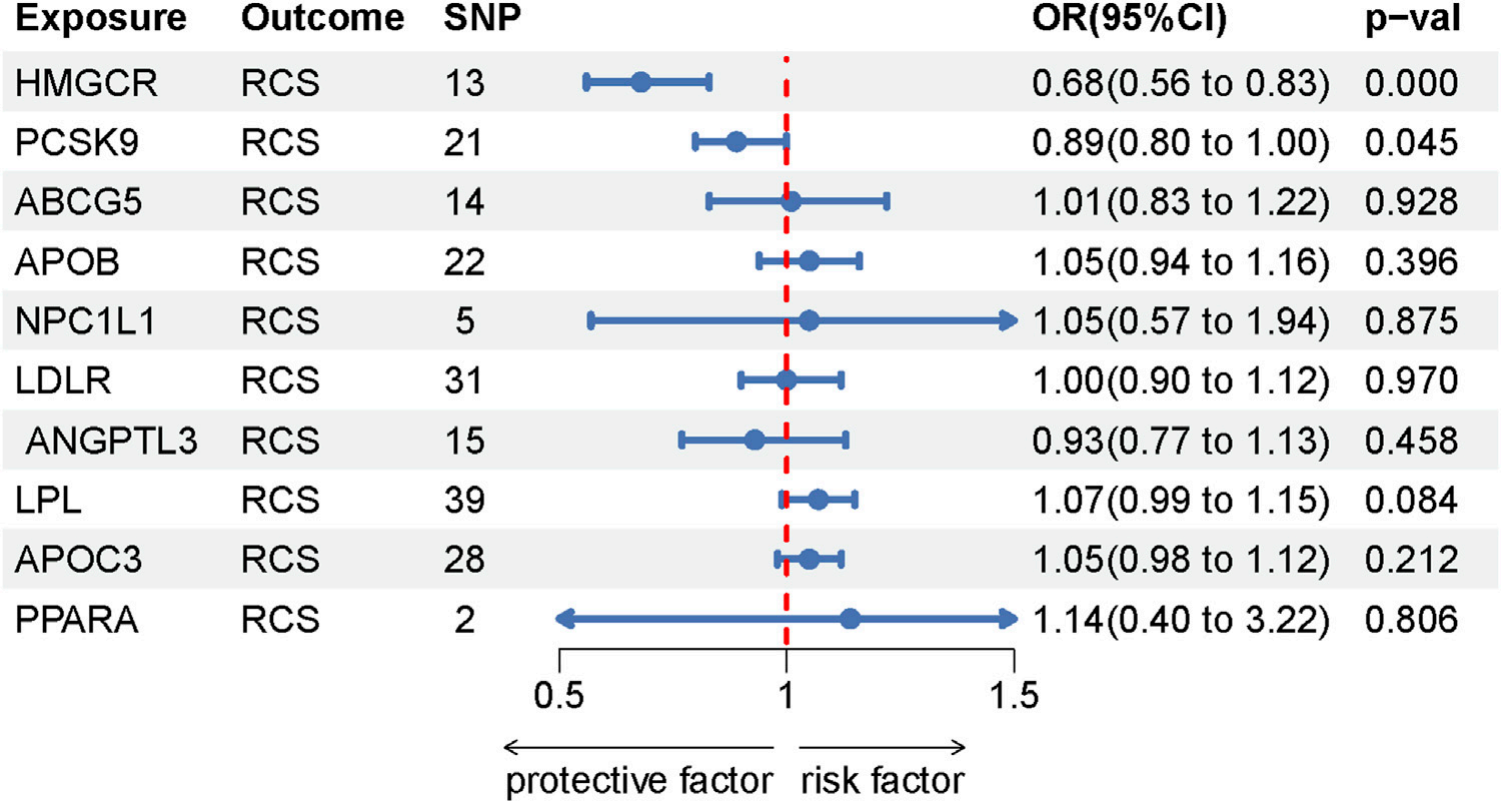
Forest plot the drug-targeted IVW method.

Moving to the study’s second segment, the analysis revealed a significant association between HMGCR inhibition and a reduced risk of RCS (OR = 0.68, [95% CI, 0.56–0.83], *p* = 1.510 × 10^−4^). In contrast, PCSK9 inhibition, while associated with a reduced risk of RCS(OR = 0.89, [95% CI, 0.80–1.00],*p* = 0.045), did not reach statistical significance. The absence of association between other drug targets and rotator cuff syndrome further narrows the focus to specific genetic interventions. Detailed MR results for these drug targets are found in Supplementary Table S9, with additional analyses on heterogeneity and pleiotropy in Supplementary Table S10. The IVW method’s Mendelian randomization results are depicted in Figure 5.

### 2.3 Gene expression and the risk of rotator cuff syndrome

**FIGURE 6:**
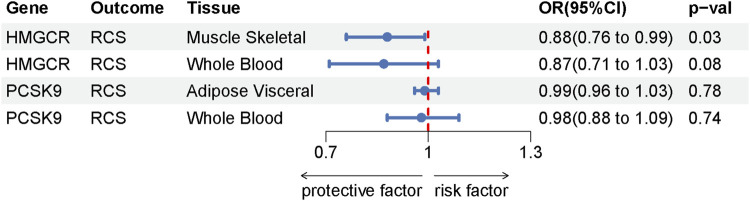
Forest plot of the SMR analysis results.

To comprehensively assess the association between positive drug target genes and susceptibility to rotator cuff syndrome, our study utilized genetic variations linked to the expression of HMGCR and PCSK9 in whole blood and various tissues as instrumental variables. We discovered that a heightened expression of HMGCR in muscle tissue correlates with a diminished risk of developing rotator cuff syndrome (OR = 0.88, [95% CI, 0.76–0.99], *p* = 0.03) However, our findings indicated no significant association between the levels of HMGCR and PCSK9 expression in whole blood, as well as PCSK9 expression in visceral fat, and the risk of rotator cuff syndrome. The results of the SMR analysis are presented in Supplementary Table S11 and the SMR forest plot is displayed in Figure 6.

### 2.4 Colocalization analysis

In our subsequent colocalization analyses, we observed a significant finding: the probability (PPH4 = 7.68%) of a causal relationship between HMGCR expression in muscle tissues and rotator cuff syndrome markedly exceeds the probability of different causal variations (PPH3 = 0.1%). Specifically, the probability of colocalization stands at an impressive 98.70% (Supplementary Table S12).

### 2.5 Mediation analysis

**FIGURE 7:**
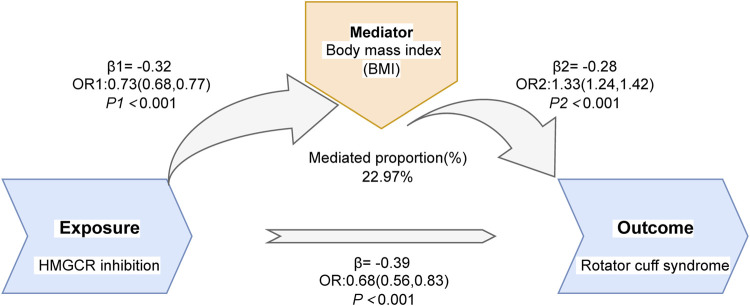
The outcome of the mediation analysis.

Given that BMI, waist circumference, and diabetes are potential risk factors for RCS, we selected these three factors for mediation analysis using Mendelian randomization. In the two-sample MR analysis of the mediator-disease relationship, we found that only BMI was a risk factor for RCS. We continued with MR analysis to evaluate the causal relationship between HMGCR inhibition and BMI, and the results indicated that HMGCR inhibition could reduce the risk of high BMI. We concluded that BMI is a mediating factor in the reduction of RCS risk through HMGCR inhibition, with a mediation effect of 23% (β1*β2/β). The results of the mediation analysis are presented in Figure 7 and Supplementary Table S13.

## 3 Discussion

Our research, which involves constructing various genetic variables, demonstrates a correlation between HMGCR inhibitors and a reduced risk of rotator cuff syndrome, thereby highlighting HMGCR as a promising therapeutic target for prevention. Additionally, three specific lipid traits and nine other lipid-lowering targets were not associated with rotator cuff syndrome.

Previous literature has reported an association between higher BMI and rotator cuff disorders. A systematic review of clinical studies conducted in 2020, which included 22 studies and 49,914 participants, demonstrated a strong positive correlation between obesity and upper limb tendon disorders (Macchi et al., 2020). Subsequently, Zhao et al. conducted a meta-analysis that included studies from 14 countries involving 9,809 subjects, and the results identified body weight as a risk factor for rotator cuff disease (Zhao et al., 2022). Our mediation analysis confirmed that BMI is a risk factor for RCS, consistent with previous reports. This suggests that a lower BMI mediates the protective effect of HMGCR inhibition on RCS.

The link between hyperlipidemia and tendon disorders, particularly rotator cuff disorders, remains a complex issue in orthopedic research. Studies have shown that individuals with rotator cuff disorders often present with higher LDL-C, TG, and TC levels (Lai and Gagnier, 2018; Yang and Qu, 2018), although contradictory reports exist that find no significant association between serum TG, TC, and these disorders (Longo et al., 2010). Recent Mendelian randomization analyses suggest a link between TC levels and rotator cuff syndrome but caution against overinterpretation due to possible confounding factors or horizontal pleiotropy (Cao et al., 2023). Our findings mirror this cautious stance, failing to definitively prove that elevated serum lipid levels are a risk factor for rotator cuff syndrome. This consistency across different studies highlights the ongoing challenge of delineating the exact role of hyperlipidemia in tendon disorders.

Statins are extensively used as cholesterol-lowering agents, primarily used for preventing and treating cardiovascular and cerebrovascular diseases. Their mechanism of action involves inhibiting the HMG-CoA reductase, which leads to a reduction in cholesterol synthesis, this reduction in cholesterol synthesis results in an increased expression of low-density lipoprotein receptors on cell surfaces, facilitating the faster clearance of serum low-density lipoproteins (Mao et al., 2022; Guo et al., 2023; Min et al., 2023). In the context of tendon diseases, statins, particularly simvastatin, have been shown to lower the risk of such disorders. A systematic evaluation reported that simvastatin reduces the risk of tendon diseases (OR = 0.62, [95% CI, 0.54–0.71], *p* < 0.001) (Teichtahl et al., 2016). Further, a comprehensive review encompassing 16 studies has indicated that the use of statins can reduce the likelihood of the development of rotator cuff disease and decrease the risk of needing revisions after rotator cuff repair surgeries (Yang and Qu, 2018). In addition to these findings, an animal study demonstrated that a 3-month course of simvastatin treatment improves the mechanical and histological properties of tendons without adversely affecting their mechanical performance, potentially lowering the risk of tendon ruptures (Tucker and Soslowsky, 2016). Additionally, cross-sectional research carried out in the Netherlands has demonstrated no marked correlation between the utilization of statins and the frequency of sports-related injuries, or injuries linked to tendons, ligaments, and muscles, thus emphasizing the safety of statins in the musculoskeletal system (Bakker et al., 2017).

In our research, we observed no link between blood lipids and rotator cuff syndrome, indicating that the beneficial effects of statins on the rotator cuff might not be related to blood lipid level reduction. However, statins’ anti-inflammatory, anti-fibrotic, and tissue regeneration properties, as highlighted in recent studies (Ko et al., 2012; Davis et al., 2015; Yang and Qu, 2018), are likely key to their efficacy in reducing rotator cuff disease risks. Notably, inflammation within the microenvironment is a significant risk factor for tendon injuries and degeneration (Shindle et al., 2011). A large cohort study has demonstrated that the use of statin medications can significantly reduce the incidence of rotator cuff disorders and aid in the treatment of inflammatory tendon diseases. This effect is likely related to their anti-inflammatory properties, with rosuvastatin exhibiting the strongest anti-inflammatory action among them (Lin et al., 2015). Blood lipids activate the NF-κB pathway, increasing levels of inflammatory markers including IL-1, IL-6, TNF-α, COX-1, and COX-2 (Hu et al., 2014; Yazdani et al., 2022). Recruiting inflammatory factors, such as TNF-α, IL-1, and IL-6, may trigger catabolic processes within intramuscular proteins, which can, in turn, result in the degeneration and fibrosis of rotator cuff muscles (Krieger et al., 2017). Preventing muscle weakness and fibrosis can effectively improve the prognosis of rotator cuff disorders. An animal study has shown that simvastatin can reduce the production of type I collagen, thereby protecting the tendon and significantly reducing fibrosis in the rotator cuff (Davis et al., 2015). Given their recognized anti-inflammatory and anti-fibrotic effects across different tissues, statins effectively protect muscles from atrophy, fibrosis, and fatty infiltration (Yazdani et al., 2022). Furthermore, extensive research indicates statins’ potential in tendon regeneration. For instance, Hao and others developed a rat model of rotator cuff injury to demonstrate that simvastatin activates the expression of vascular endothelial growth factor (VEGF) in osteoblasts through the phosphoinositide 3-kinase (PI3K) signaling pathway, thus aiding in the healing of the rotator cuff. Additionally, simvastatin-infused silk fibroin promotes the proliferation and differentiation of bone marrow stem cells through beta-catenin signaling, specifically enhancing tendon-bone healing, increasing collagen production, and improving the biomechanical integrity of the rotator cuff (Hao et al., 2021). Dolkart (Dolkart et al., 2014) used a rat model to examine the impact of statins on rotator cuff injuries. They discovered that atorvastatin enhances COX-2 activity and autocrine/paracrine PGE2 signaling, thereby promoting the proliferation, migration, and adhesion of tendon cells and facilitating tendon repair following rotator cuff surgery.

Our research indicates that statin lipid-lowering drugs can help reduce the risk of rotator cuff syndrome. However, most current investigations into their specific mechanisms predominantly focus on animal models and lack depth, with a continued absence of high-quality studies. Given these facts, future research should emphasize further investigations into cellular and molecular mechanisms (Qian et al., 2023). While drug-target Mendelian randomization studies can reveal causal relationships between medications and diseases, we still believe that high-quality clinical research is essential.

There are some limitations to this study. First, Mendelian randomization only provides the direction of correlation between exposure and outcomes, without quantifying the extent, and it cannot substitute for clinical trials, requiring further clinical trials to assess the connection between blood lipids and rotator cuff syndrome. Additionally, as the UK Biobank lacks GWAS data for TC, this data was obtained from the Global Lipids Genetics Consortium (GLGC). Therefore, the lipid trait data originating from two separate institutions may increase bias to some extent. Finally, as all data in this study come from European ethnicities, the findings might not apply to populations in other areas.

## 4 Conclusion

Our research does not support low-density LDL-C, TG, and TC as risk factors for rotator cuff syndrome. Furthermore, the study identifies HMGCR as a potential pharmacological target for preventing and treating rotator cuff syndrome.

## Data Availability

The original contributions presented in the study are included in the article/Supplementary Material, further inquiries can be directed to the corresponding author.

## Supplementary material

SUPPLEMENTARY FIGURE S1 Scatter plot of the causal association between drug target and RCS using different MR methods. **(A)** HMGCR **(B)** PCSK9.

SUPPLEMENTARY FIGURE S2 Funnel plot of the causal association between drug target and RCS. **(A)** HMGCR **(B)** PCSK9.

SUPPLEMENTARY FIGURE S3 Forest plot of the causal association between drug target and RCS. **(A)** HMGCR **(B)** PCSK9.

SUPPLEMENTARY FIGURE S4 Leave-one-out sensitivity analysis of the causal effect of drug target on RCS. **(A)** HMGCR **(B)** PCSK9.
