# Supplementary figures and images for: A causal association between lipid-lowering medications and rotator cuff syndrome: a drug-targeted mendelian randomization study
Source: Front Genet. 2024 Jun 6;15:1383646. doi: 10.3389/fgene.2024.1383646 (PMC11187090; doi:10.3389/fgene.2024.1383646)

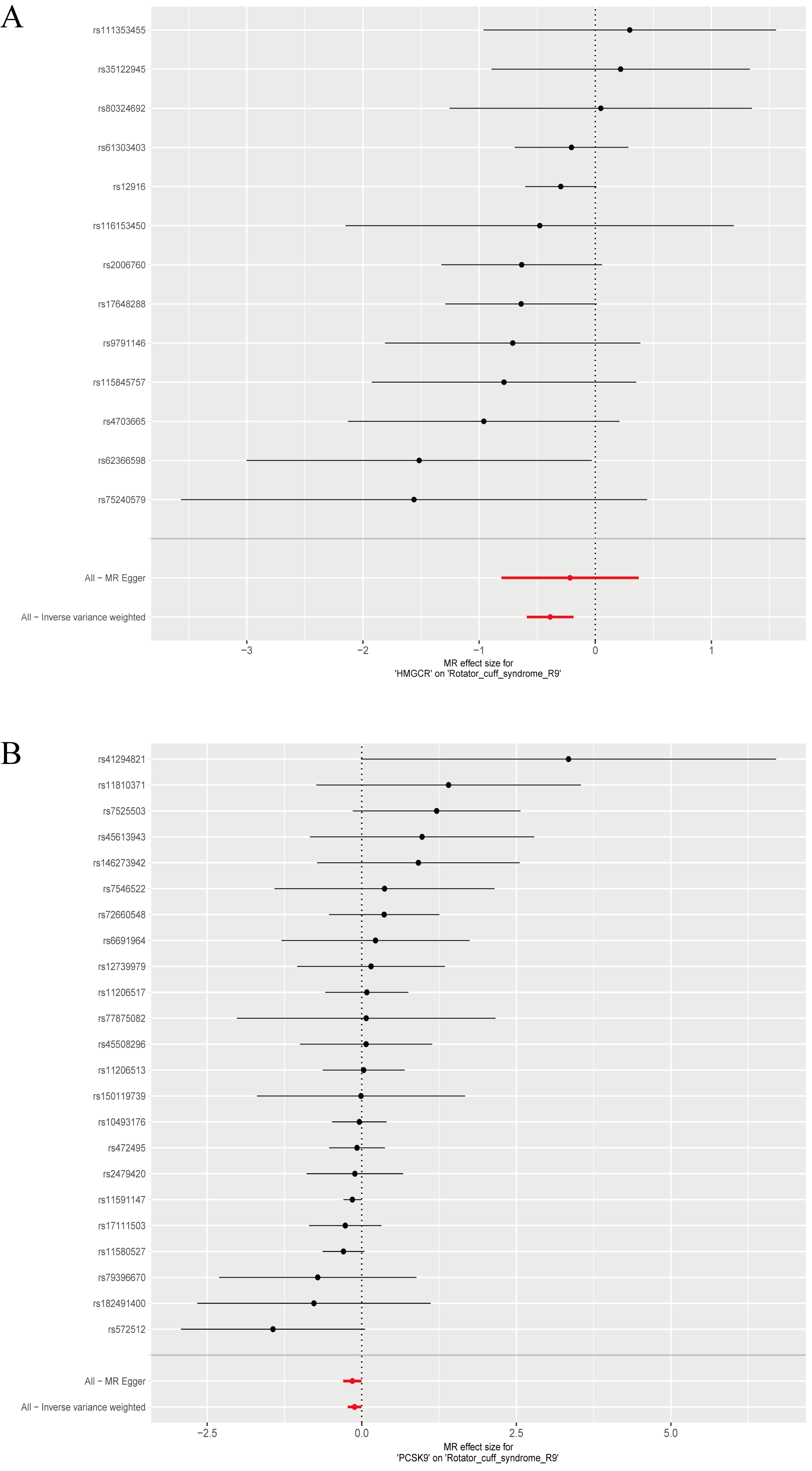

Supplement: Supplementary file 3 [file Image3.TIF]

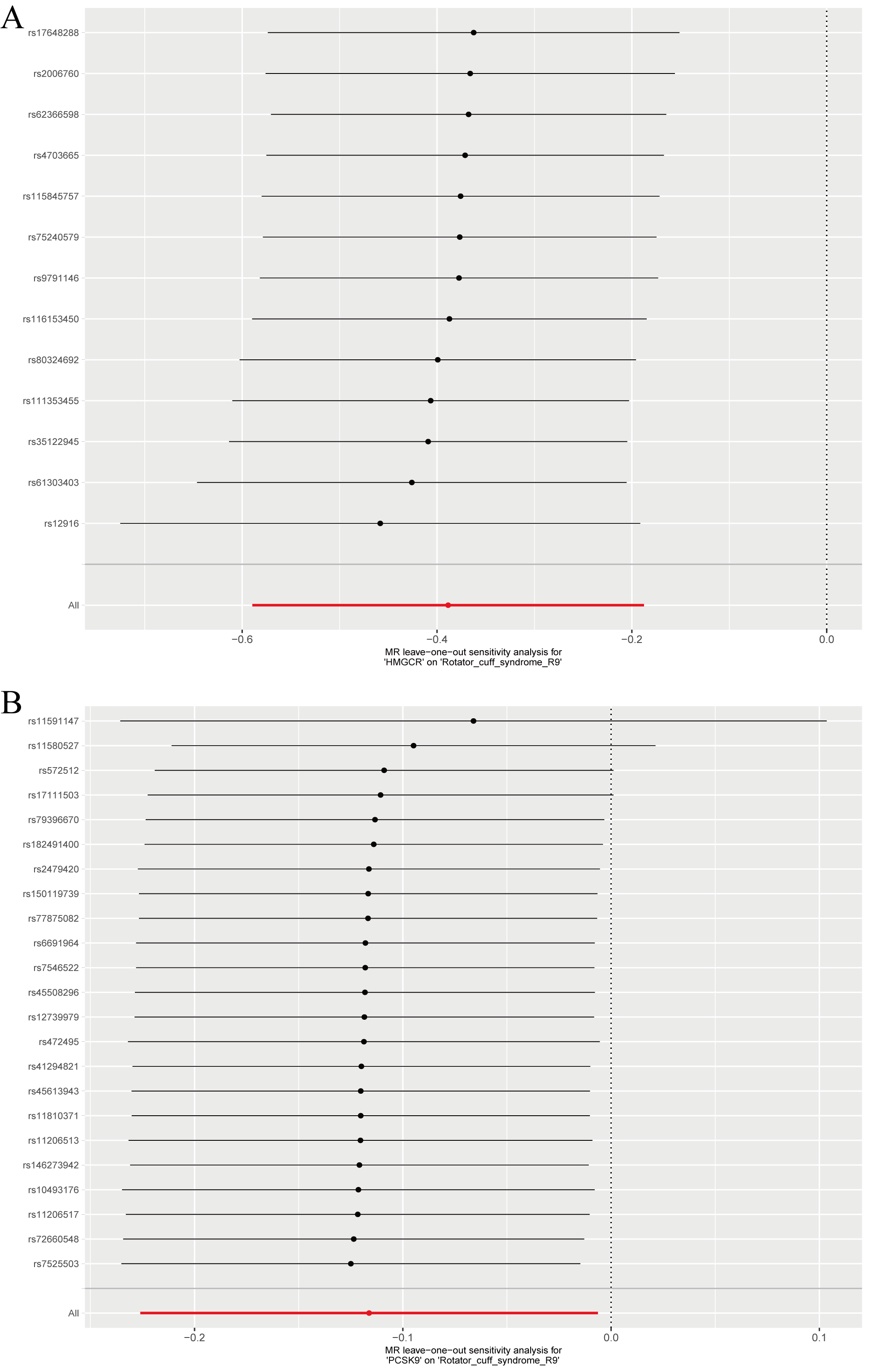

Supplement: Supplementary file 4 [file Image4.TIF]

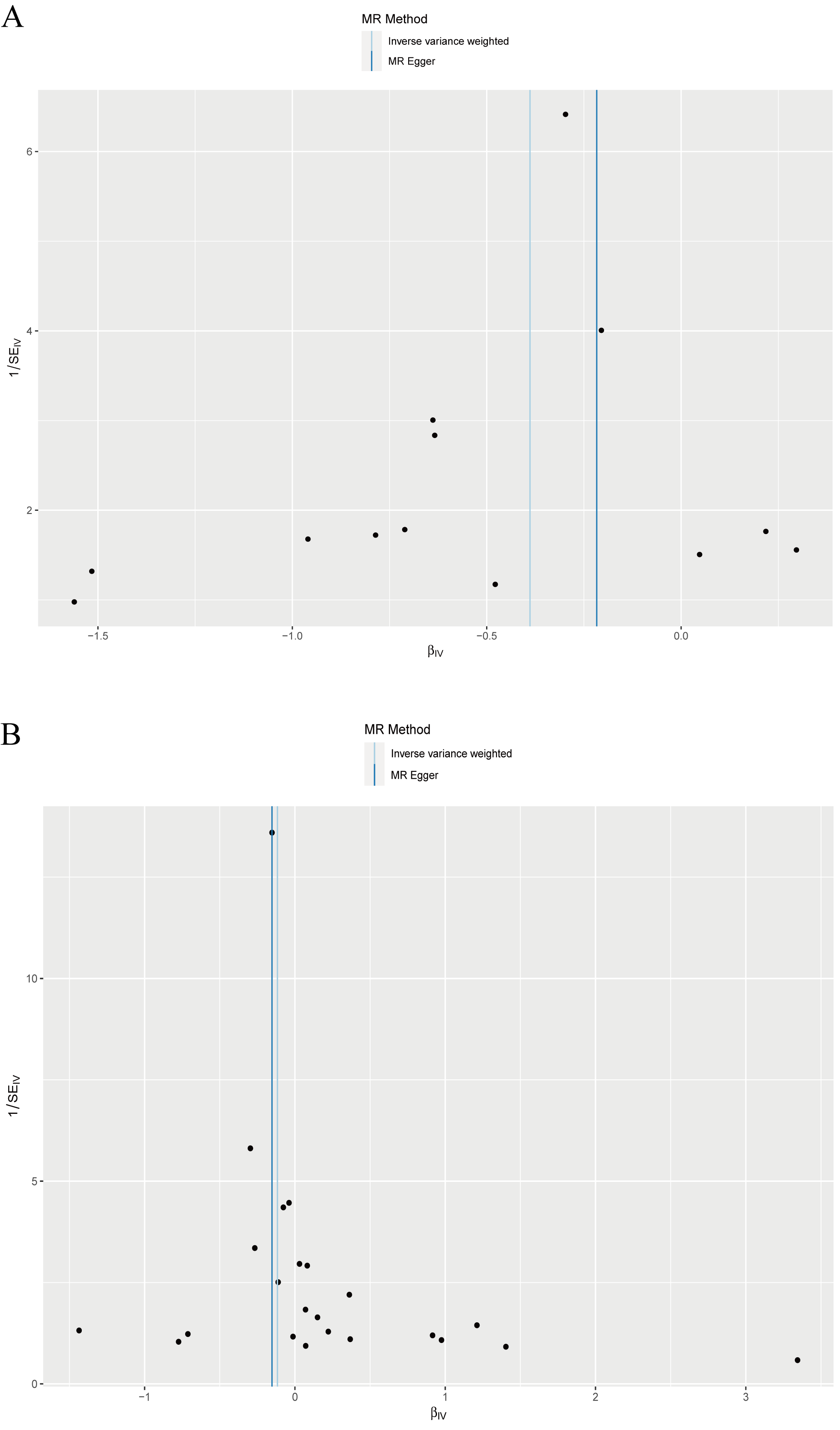

Supplement: Supplementary file 5 [file Image2.TIF]

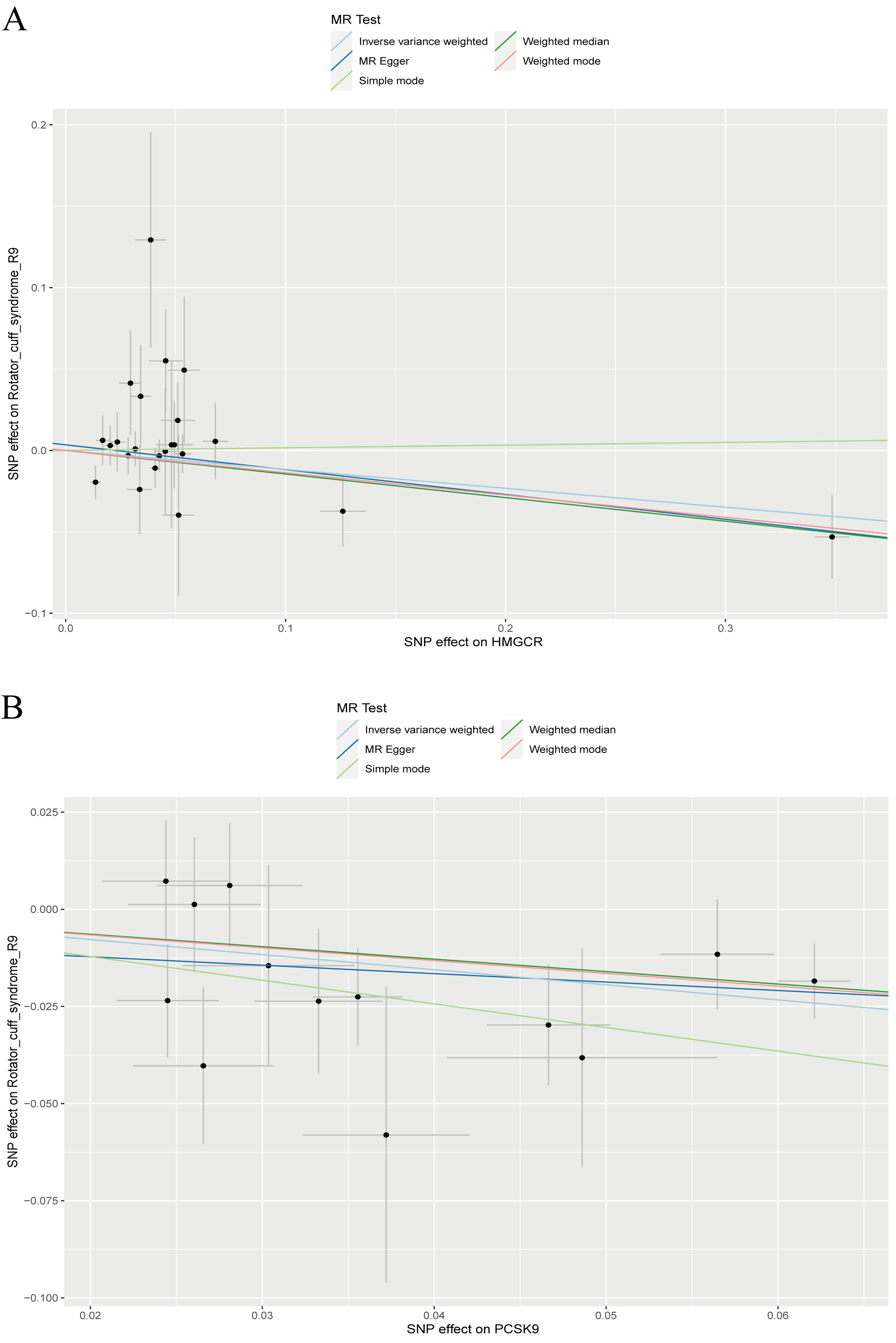

Supplement: Supplementary file 6 [file Image1.TIF]
